# Supplementary material for: Orthodontic Status and Association with Oral-Health-Related Quality of Life—A Study of 16-Year-Old Norwegians with a Cleft Lip and Palate
Source: Int J Environ Res Public Health. 2024 Apr 26;21(5):550. doi: 10.3390/ijerph21050550 (PMC11121370; doi:10.3390/ijerph21050550)
Supplement: Supplementary file 1 [file ijerph-21-00550-s001.zip › ijerph-2942539-supplementary.pdf]

Orthodontic Status and Association with Oral-Health-Related Quality of Life—A Study of 16-Year-Old Norwegians with a Cleft Lip and Palate

Supplementary file

Table S1. Bergen Orthodontic Grading Index

**Intermaxillary Sagittal relation (ANB-angle/facial profile)**
1 Poor (ANB= <-5°)
2 Rather poor (ANB= ≥ -5°and<-2°)
3 Fair (ANB= ≥-2°and<0°)
4 Good (ANB = ≥0°and≤2°)
5 Perfect (ANB = >2°)
**Dental arch and future need for orthodontic and prosthodontic treatment**
1 Major spacing/missing teeth
2 Minor spacing/missing teeth
3 Misaligned teeth
4 Acceptable aligned dentitions
5 Perfect aligned dentitions
**Occlusion and the need for orthognathic surgery**
1 Negative overjet more than 8 mm
2 Negative overjet -1 to -8 mm
3 Edge to edge occlusion
4 Positive overjet
5 Perfect occlusion and overjet

Table S2. Frequency distribution n (%) of clinical indicators, according to cleft type.

Clinical indicators	UCLP n (%)	BCLP n (%)	CL n (%)	CP n (%)	Total n (%)
**Missing teeth (agenesis or extracted due to poor quality)**					
Yes	27 (84.4) *	11 (100) *	11 (32.4)	17 (37.8)	66 (54.1)
No	5 (15.6)	0 (0)	23 (67.6)	28(62.2)	56 (45.9)
**Intermaxillary sagittal relations (facial profile)**					
ANB= <0º (poor – fair sagittal relationship)	21 (65.6) *	7 (63.6) *	11(32.4)	3 (6.7)	42 (34.4)
ANB= ≥0º (good – perfect sagittal relationship)	11(34.4)	4(36.4)	23(67.6)	42 (93.3)	80 (65.6)
**Dental arch**					
Spacing and malaligned dentition	18(56.3) *	9 (81.8) *	6 (17.6)	6 (13.3)	39 (32.0)
Acceptable-perfect dentition	14(43.8)	2 (18.2)	28 (82.4)	39 (86.7)	83 (68.0)
**Occlusion**					
Negative overjet or open bite	9 (28.1) *	4 (36.4) *	1 (2.9)	3 (6.7)	17(13.9)
Positive overjet and good occlusion	23 (71.9)	7 (63.6)	33 (97.1)	42 (93.3)	105(86.1)

*p<0.05

Table S3. The unadjusted association between satisfaction with dental aesthetic and OHRQoL.

	Self-reported dental aesthetic	
OHIP-14 scoring	Satisfied	Not satisfied
No impact (OHIP-14=0)	23 (23.5)	1 (4.2)
Impact (OHIP-14>0)	75 (76.5)	23 (95.8)*
Total	98 (80.3)	24 (19.7)

*p<0.05
